# Supplementary material for: Comprehensive at-arrival transcriptomic analysis of post-weaned beef cattle uncovers type I interferon and antiviral mechanisms associated with bovine respiratory disease mortality
Source: PLoS One. 2021 Apr 26;16(4):e0250758. doi: 10.1371/journal.pone.0250758 (PMC8075194; doi:10.1371/journal.pone.0250758)

Figure 1: Top hits against known bovine virus sequences for S_33

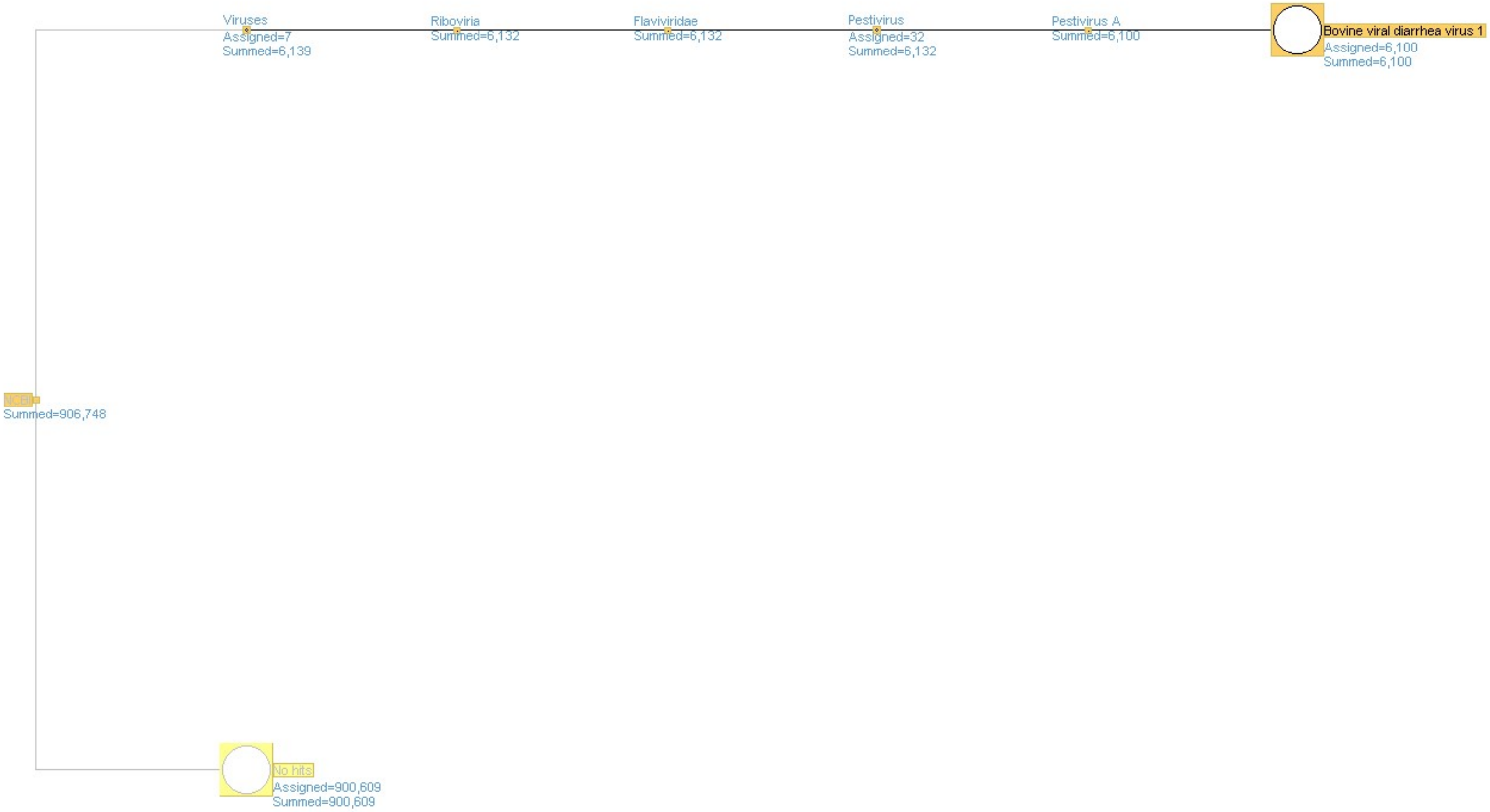

Figure 2: Top hits against known bovine virus sequences for S_51

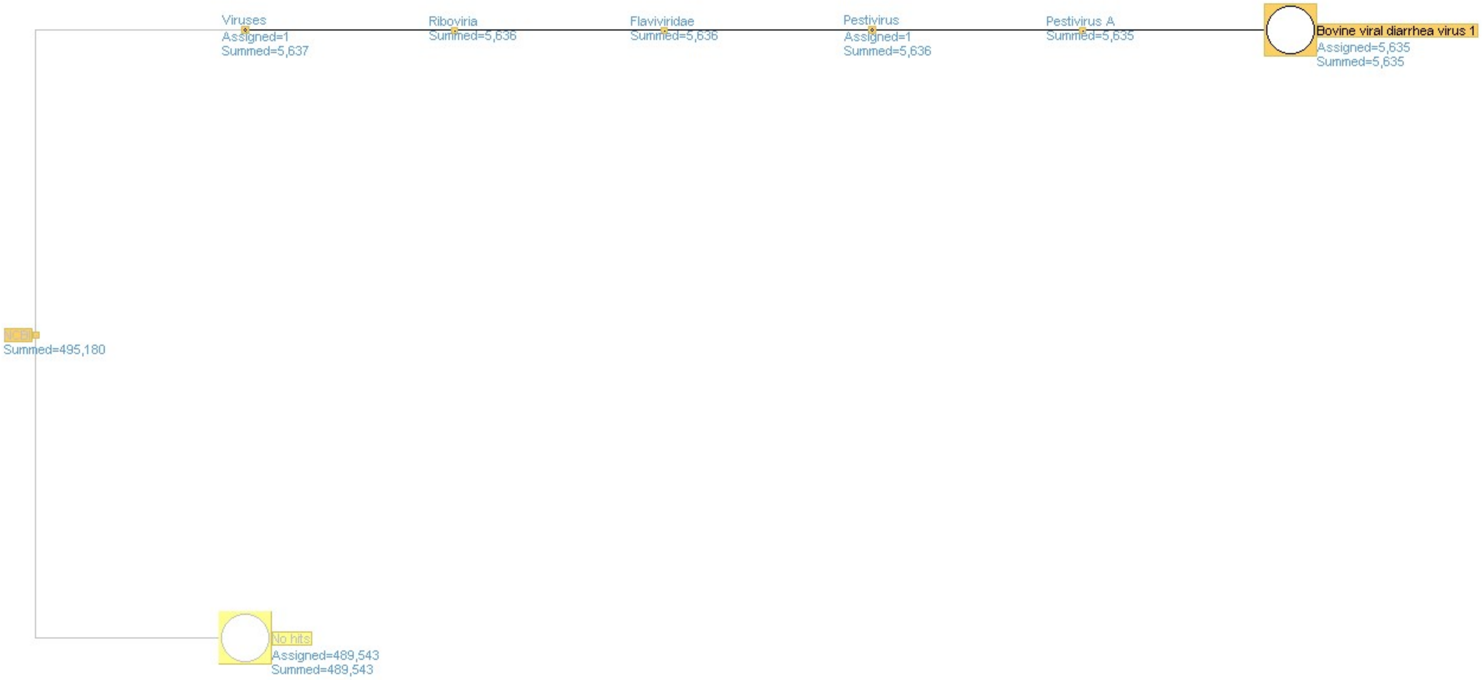

Figure 3: Top hits against known bovine virus sequences for S_52

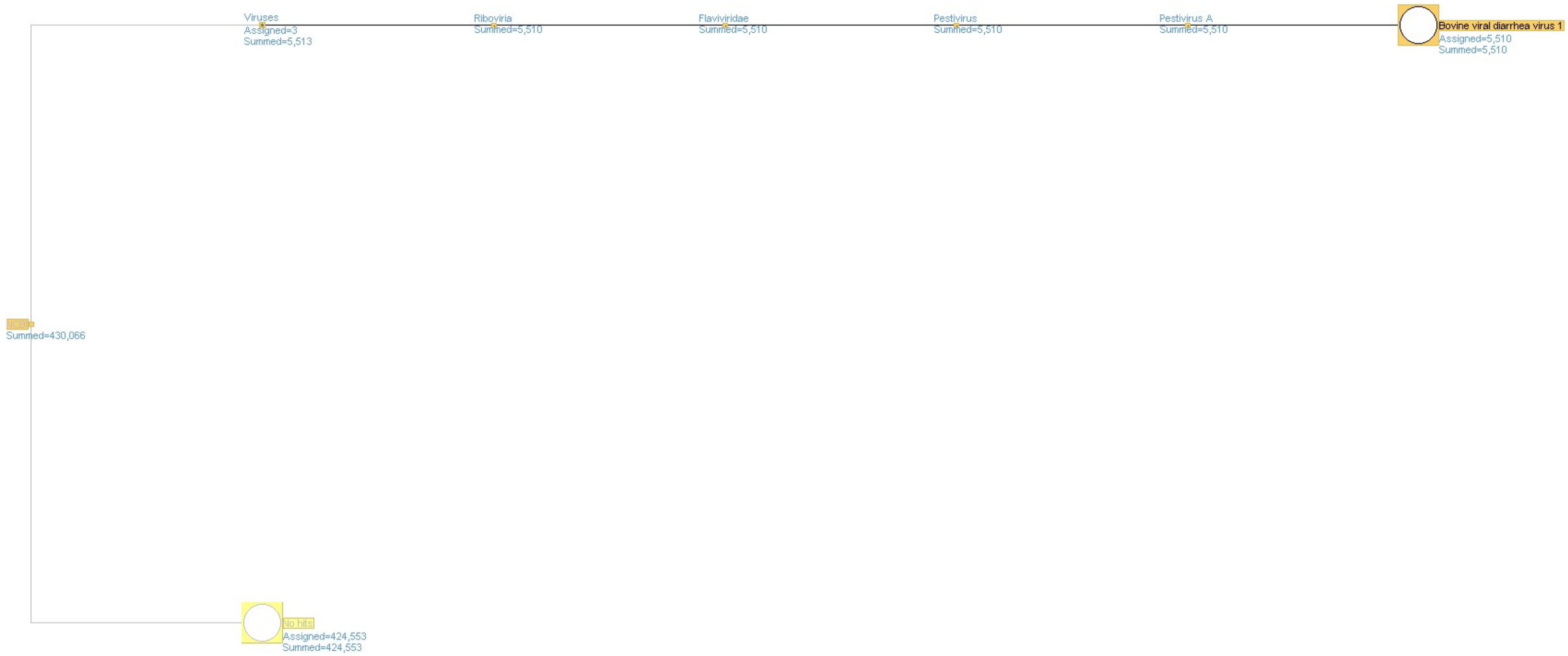

Figure 4: Top hits against known bovine virus sequences for S_75

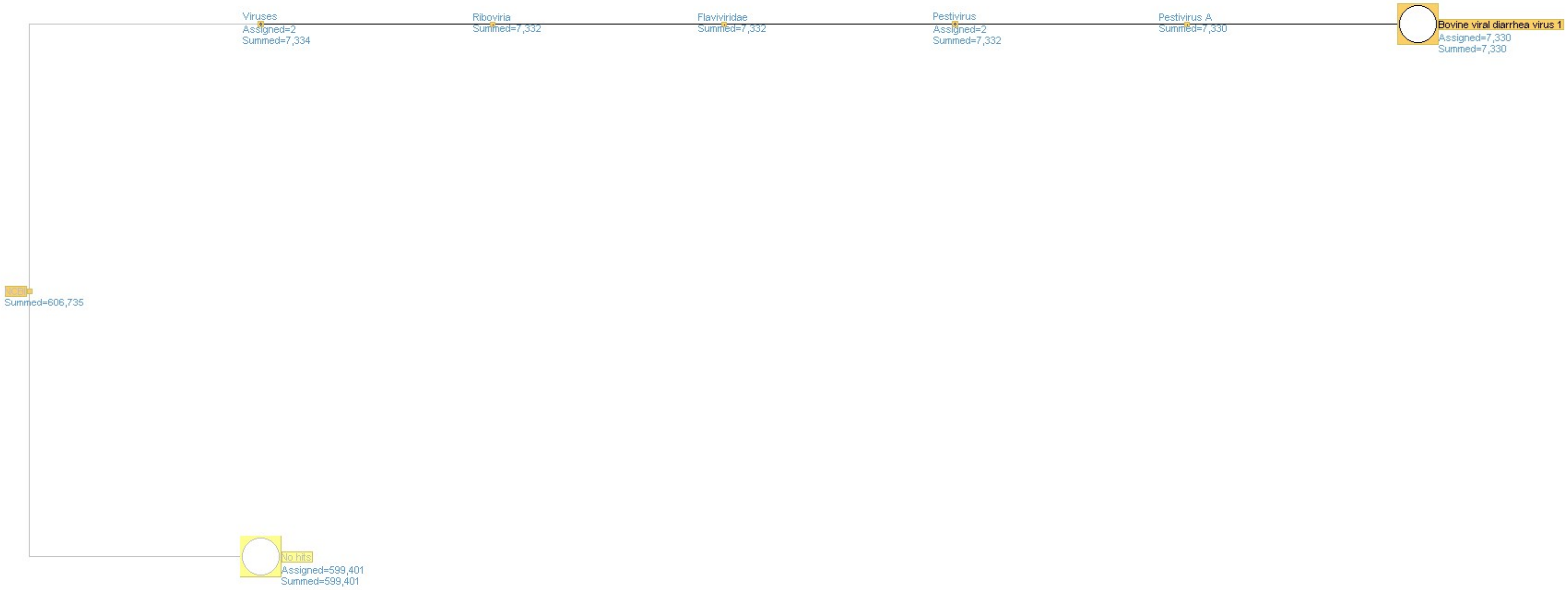

Figure 5: Top hits against known bovine virus sequences for S_76

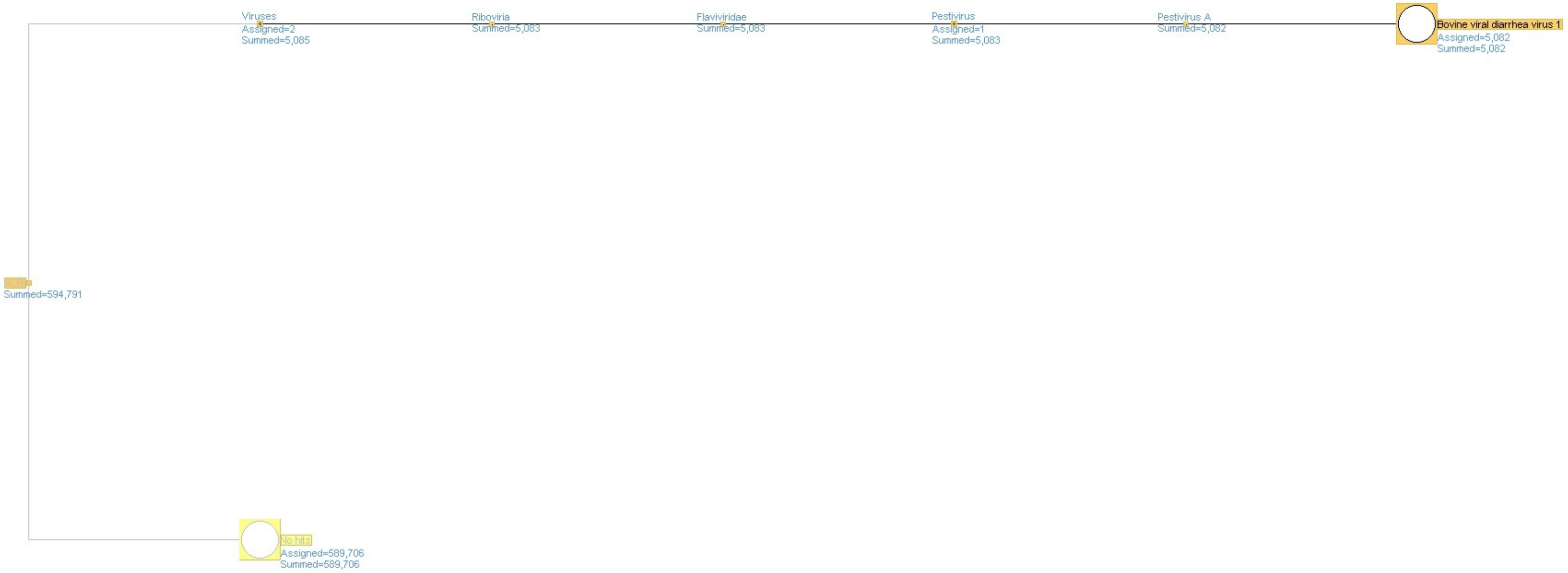

Figure 6: Top hits against known bovine virus sequences for S_85

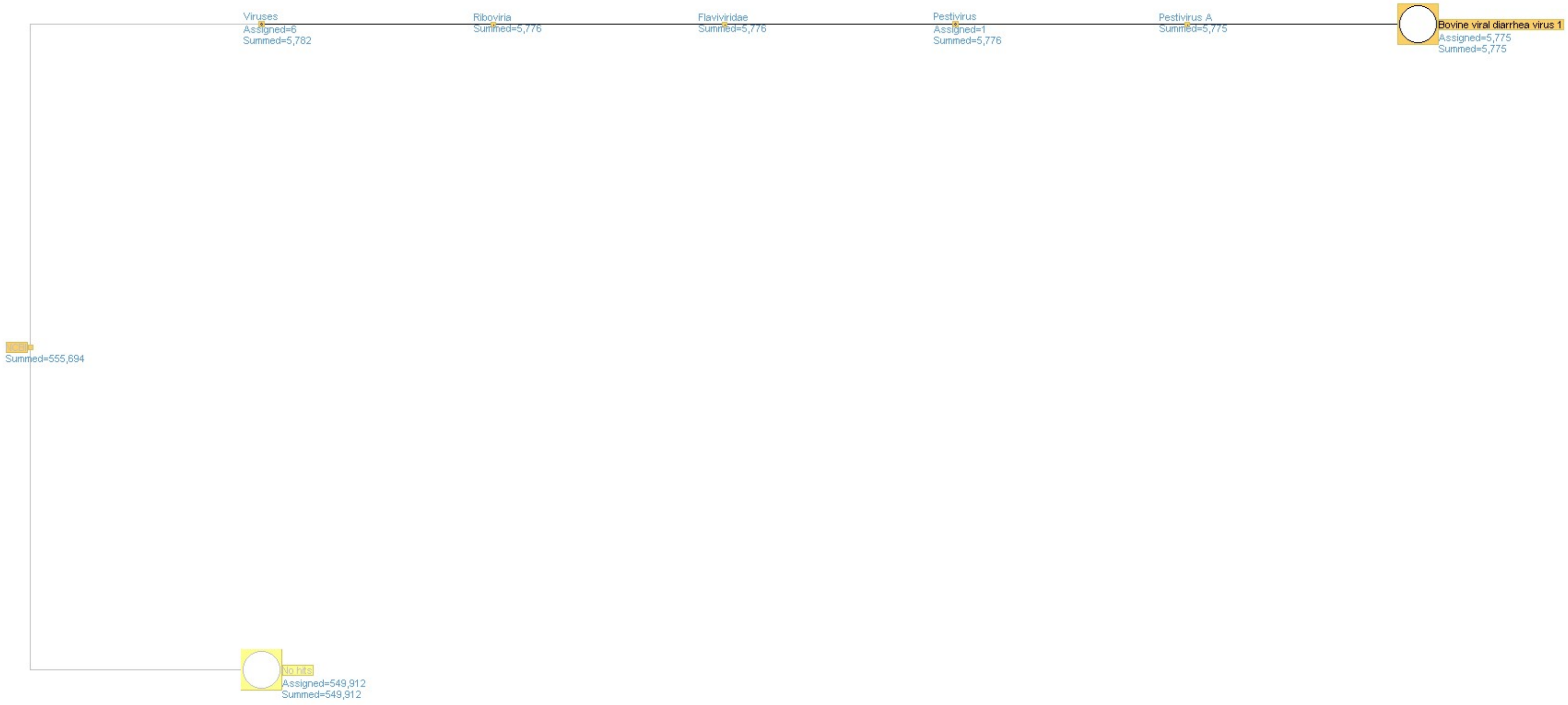

Supplement: S5 File — (PDF) [file pone.0250758.s005.pdf]
