# Supplementary material for: Comprehensive at-arrival transcriptomic analysis of post-weaned beef cattle uncovers type I interferon and antiviral mechanisms associated with bovine respiratory disease mortality
Source: PLoS One. 2021 Apr 26;16(4):e0250758. doi: 10.1371/journal.pone.0250758 (PMC8075194; doi:10.1371/journal.pone.0250758)
Supplement: Attachment — Submitted filename: Response to Reviewers.docx [file pone.0250758.s007.docx]

Mississippi State University

College of Veterinary Medicine

P.O. Box 6100

240 Wise Center Drive

Mississippi State, MS 39762

To the Editor,

We thank the reviewers for their comments regarding our manuscript (PONE-D-20-27349). We have addressed the comments and have made revisions accordingly. Our responses have been addressed in a bullet point manner below, and all changes are conveyed through Microsoft Word Track Changes in the “Revised Manuscript with Track Changes” document. Line numbers indicated in our responses below refer to the version of the revised manuscript without changes tracked.

Again, we thank you for your time and effort made and hope that our revised version is now acceptable for publication in PLOS ONE.

Sincerely,

Dr. Matthew A. Scott

On behalf of all authors.

**Response to Reviewer 1:**

General comments:

1. *While I understand the limitations of a project such as this, only 6 animals were evaluated in this study - 3 dead and 3 alive. How do the authors think that this small sample size might have impacted outcome? Would the results be different if a larger number of animals were included? Could some comment regarding this be placed in the discussion?*
   - We appreciate and understand this concern regarding sample size and statistical power. It is generally accepted that increasing the sequencing depth will increase statistical power, albeit around 20-40M reads/sample before incurring minimal gains, and that sample size has a greater impact on statistical power than sequencing depth. Obtaining ­*a priori* sample size calculations remains a contested and developing aspect of RNA-Seq, due to the heterogeneous and non-normal distribution of data, multiple hypothesis testing and correction, and lack of prior expression knowledge based on the study (pilot data).
   - The predominant impact of including a larger number of animals in this study would be an overall decrease in expected type II error rates, assuming no changes in the biological coefficient of variation. It is worth noting that many of the RNA-Seq packages utilized, including edgeR, are optimized for experiments using a limited number of biological replicates per group. Additionally, it is generally concluded that three biological replicates per group is the minimum number necessary for meaningful inferential analysis.
     - References to these responses: DOI: 10.1093/bib/bbw144; DOI: 10.1186/s12859-018-2445-2; DOI: 10.1261/rna.053959.115; DOI: 10.1089/cmb.2012.0283; DOI: 10.1186/s13059-016-0881-8
   - Lines 288-294 have been included to briefly illustrate the preceding points.
2. *Abstract – lines 24-25: The statement beginning with "Clinical diagnosis" is somewhat confusing. While I understand the intent, visual detection methods are both non-specific and insensitive. It is stated more clearly in the introduction (Lines 60-61).*
   - Lines 24-25 were edited to clarify concerns regarding BRD diagnostics.
3. *Discussion – the results of the study would suggest that animals that succumb to the effects of BRD are potentially combating viral pathogens at the time of facility arrival. How do the authors see the results of this study being used in the management of high-risk beef stocker calves to reduce antimicrobial use and improve survival? What would a future direction for this work entail? In other words, how could this data be used in the future at a herd level?*
   - Lines 253-258 were added to address these comments.

**Response to Reviewer 2:**

1. *Line 56: I'm not familiar with the term “newly placed,” perhaps recently transported?*
   - Line 56 was edited to change the term “newly placed” to “recently transported”.
2. *Line 75: This sentence is not quite right. Too many “to”s and you need to compare something to something else.*
   - Lines 75-78 were addressed to remove ambiguity and improve sentence structure.
3. *Line 110: Does this mean that these 69 genes were differentially expressed between ALL the dead and all the alive, and any differences within each group were not included? For example, if DEAD 1 and 2 upregulated gene A, but not DEAD 3, was this gene included or not?*
   - Correct, those 69 genes were significantly differentially expressed between the two groups, not in individuals. The study design of this project was developed to assess differences between groups and not individual animals. The normalization technique (trimmed mean of M-values) allows for inter-sample comparison of relative gene abundances, where genes from the two groups were evaluated for significance with likelihood ratio testing. For example, if gene A possessed a large enough variance within the DEAD cohort due to one animal having a considerably different relative abundance (post-normalization), the scaled ratio of the group would have a meaningful change and most likely effect significance and relative fold change when compared to the ALIVE group.
4. *Line 164 (Figure 3): These figures are incredibly hard to read. The colors in figure A are difficult to differentiate, especially with the interconnected lines, and the reflection on the bubbles is distracting and complicates the image needlessly. In figure B it is difficult to see the lighter pink squares. I would also strongly recommend checking with guidelines for color blind friendly coloring in figures.*
   - We acknowledge the comments regarding accessibility in scientific figures. Regarding Figure 3, these network images are generated by the STRING database interface and do not allow for manipulations without distorting the figure. The coloring seen within the protein nodes represent that they are primary (1^st^ shell) interactors and the icons seen within them depict that there is a known structure associated with the protein. While we agree that the bitmap reflections seen across each of these nodes may be unnecessary, they cannot be removed without manipulating the image file.
   - Regarding coloration, the color mapping within the file has been adjusted to better represent the image for individuals with deuteranomaly.
5. *Line 243: This seems like a fairly strong statement without any actual evidence of infection?*
   - Line 242 was adjusted to convey a less direct statement. While our study did not include viral isolation methods at arrival, nor identified viral reads that could relay direct causation of the type I interferon response, several studies cited within the paragraph have identified the same genes, in expressional directionality and relative abundance, in animals both experimentally and naturally infected with viruses.
6. *Line 304: How often are cattle viremic for BRD pathogens? Would you expect to find viral transcripts if the animals are indeed infected at arrival?*
   - The likelihood of viremia varies for different BRD agents; e.g., viremia is likely in BVDV infection but not in BRSV infection. It should be noted that common viral detection assays, such as RT-PCR, are less sensitive for detection of nucleic acids, compared to sequencing methodology. Additionally, with the exception of BVDV, viral detection assays are rarely employed in beef production settings. Therefore, it may be hypothesized that viremia is an underreported phenomenon in BRD pathogenesis and may be detected with RNA-Seq methodology.
   - 3’ polyadenylated tails are a common feature in positive-strand RNA and some dsDNA viruses. Viruses such as bovine coronavirus, BVDV, and PI-3V possess polyadenylated tails and have been shown to cause viremia. Thus, it is theoretically possible to detect rare sequenced reads with a poly-A tail dependent library preparation employed in this experiment. Impairment of viral clearance or persistent infection (i.e. BVDV) might allow for viremia to occur in high-risk cattle around the time of arrival. Thus, it is possible that viral transcripts could be detected if the animals were infected at arrival with a virus capable of causing viremia, the cattle possessed impaired clearance mechanism, and samples were collected at the right time.
     - References to these responses: DOI: 10.1007/s00705-007-1005-2; DOI: 10.1186/s12859-018-2246-7
7. *Line 324: This conclusion seems to state that these candidate genes provide an early detection method for BRD, however this study did not address that question. Also, if this is the statement the authors wish to make, it would be useful to compare this with early pathogen detection techniques which would be cheaper and more practical at arrival.*
   - We agree and appreciate the comment that other experiments should utilize the information presented for assessment of these antiviral genes at arrival in comparison with current pathogen detection techniques. The development of such an assay could allow for more accurate and rapid detection of viral disease and the generation of novel management techniques. Lines 334-337 have been included in the conclusion to address this.
